# The effectiveness of two types of MADS for OSA therapy

**DOI:** 10.1007/s00784-017-2290-0

**Published:** 2017-12-06

**Authors:** Francis Emma Verburg, Klara Hilda Alphonsine Bollen, Henk-Jan Donker, Gerbrand Johannes Cornelis Kramer

**Affiliations:** 1 Dentist cabinet Egmond, 1931 GB Egmond aan Zee, the Netherlands; 2 Department of Orthodontics, Academic Centre of Dentistry Amsterdam (ACTA), Floor 4, Gustav Mahlerlaan 3004, 3004 1081 LA Amsterdam, the Netherlands; 3 Department of Orthodontics, North West Clinics, Medical Centre Alkmaar (MCA), Wilhelminalaan 12, 1815 JD Alkmaar, the Netherlands

**Keywords:** Obstructive sleep apnea, Mandibular advancement device, Mandibular repositioning appliance, Apnea, Apnea-hypopnea index, Snoring

## Abstract

**Objectives:**

The purpose of this study was to determine differences in effectiveness between two types of mandibular advancement device (MAD).

**Material and methods:**

In this retrospective, cohort study, the two devices used were MAD type “Somnodent-Flex” (MAD 1) and MAD type “Herbst” (MAD 2). One hundred thirty-seven patients participated in this study, 67 patients were treated with MAD 1, and 70 patients with MAD 2. The indication MAD with obstructive sleep apnea (OSA) is based on a polysomnography test, in accordance with the CBO guidelines. The effectiveness of MAD therapy can be determined by a second polysomnography test (with the MAD in situ). The apnea-hypopnea index (AHI) is registered during the first and the second polysomnography test. Changes in these values determine the effectiveness.

**Results:**

A significant decrease in AHI was found regarding T1 and T2 for both the MADs: *F* (1, 134) = 140,850, *p* < 0,001. The mean differences of both the MADs turned out to correlate to T1. After correcting for this covariance, there was no significant difference between the two MAD devices regarding the AHI value: *F* (1, 134) = 1160, *p* = 0,283.

**Conclusions:**

The results of the present study show no significant difference in effectiveness between MAD 1 and MAD 2 in respect to the AHI value.

**Clinical relevance:**

Since 2012, healthcare insurance companies in the Netherlands refunds MAD type “Somnodent” used for treatment of sleep apnea. It is important to investigate if this type of MAD is as more effective or less effective as other types of MADs. If research points out that other MADs are more effective in reducing the sleep apnea, refund policies have to be adapted.

## Introduction

Sleep apnea is characterized by snoring, oxygen desaturations, and frequent arousals [1]. Sleep apnea can be divided into central-, obstructive-, mixed-, and position-dependent sleep apnea [2]. During sleep, modifications of pharyngeal muscle tone take place that lead to airway narrowing and hypoventilation. In case of individuals who already have anatomically narrowed upper airways, this can lead to partial airway closure (hypopnea) or total obstruction (apnea) [3].

Patients with obstructive sleep apnea (OSA) suffer from excessive fatigue during daytime, morning headaches [1], impaired quality of (social) life [4, 5], longer reaction times, and short-term memory loss [6, 7]. The severity of OSA is based on the subjective symptoms of sleepiness and the measured apnea-hypopnea index (AHI) (objective) [8].

Polysomnography is the golden standard to demonstrate OSA. Simultaneous airflow and oxygen saturation measurements are registered [2]. Population-based studies from the USA, Europe, and Australasia estimate a prevalence of approximately 3–7% in middle-aged males and 2–5% in middle-aged females [9].

Continuous positive airway pressure (CPAP) is defined as the standardized way to treat OSA [10]. Despite its high therapeutic efficacy, CPAP is an intensive treatment method which patients not always seem to endure. This often results in reduced clinical effectiveness [11, 12]. For patients with mild-to-moderate sleep apnea, treatment with oral appliances, like a mandibular advancement device (MAD), are considered a valid option. This also applies to patients who do not cope with or refuse CPAP treatment [13–15].

The airway passage can be improved during sleep by displacing the mandible, tongue, and pharyngeal structures with these oral appliances. Although there are several types of oral appliances, the MAD is the most commonly applied device. The MAD fixates the mandible in a forward (pronated) position [14, 16].

Originally in the Netherlands, all types of MADs were refunded and the clinician could therefore choose any type of MAD. Because of a change in the healthcare refunding policy from 2012 onwards, the insurance companies determined which type of MAD was to be refunded. As a result, only the MAD “type Somnodent-Flex” was fully refunded by the insurance companies. Therefore, this type of MAD came into use during the years 2012–2014.Whether the latter MAD is the most effective is not proven. The difference in effectiveness can be defined by the difference in AHI value. The AHI value is measured during two polysomnography tests. The first polysomnography test is performed to determine OSA. OSA is defined by an AHI value of > 10. When OSA is determined, patients can be treated with a MAD device, respectively, MAD 1 or MAD 2. After wearing the MAD device for at least 3 months, patients return for a second polysomnography test (with the MAD in situ).

The purpose of this study is to determine if there is a difference in effectiveness between two types of MAD devices. The two devices used are MAD type “Somnodent-Flex” (MAD 1) and MAD type “Herbst” (MAD 2). The effectiveness of the MAD is defined by the difference of the AHI value between the first and the second polysomnography test.

In this study following hypothesis has been tested:

### H_0_:

There is no significant difference in effectivity between both MRA’s, regarding the AHI value.

### H_1_:

There is a significant difference in effectivity between both MRA’s, regarding the AHI value.

## Material and methods

### Patients

According to the STROBE guidelines, this observational cohort study included OSA patients treated at the North-West Clinics Alkmaar (formerly Medical Centre Alkmaar) from 2008 to 2014. The patients consulted their family doctor or dentist with complaints such as snoring, sleepiness during daytime, and arousals. They were referred to the apnea team that consists of different medical specialists like an ear, nose, and throat specialist (ENT-specialist), a neurologist, or a pulmonologist for further examination.

OSA patients are treated as indicated by the Dutch Apnea guidelines determined by the CBO [2]. The AHI stands for number of respiratory events per hour for at least 10 s per event [2]. Patients with an AHI greater than 30 are treated with a CPAP and no MAD. Only in cases when CPAP is not tolerated by the patient, MAD can be a second treatment option. Therefore, we did not include patients with comparable AHI values but treated with a CPAP.

The need for treatment is based upon a polysomnography test. During this sleeping test, AHI values among others are registered. When the polysomnography test confirms OSA, an endoscopy is performed in order to determine the level of obstruction. If the obstruction is situated in the upper airways, the patient is considered suitable for treatment with an MAD and is referred to the orthodontic department.

Patients were referred for a second polysomnography test 3 months after MAD placement. After this referral, the second polysomnography test (with the MAD in situ) was registered within 6 to 8 weeks. AHI is registered during the first and the second polysomnography test. Changes in these values determine the effectiveness. MAD effectiveness is defined as: the difference in AHI before and after 3 months of MAD treatment.

**Fig. 1:**
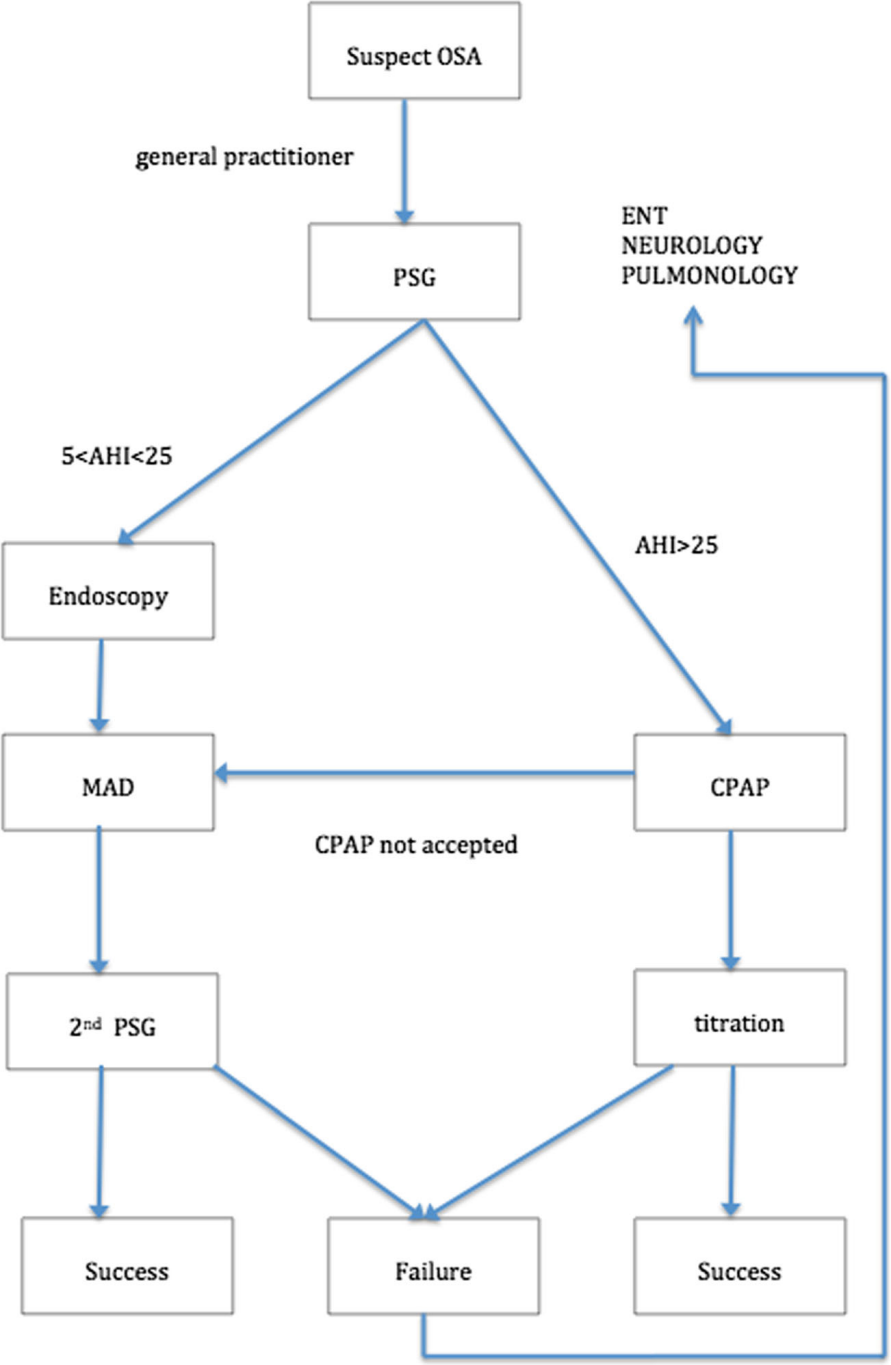
Flowchart concerning patient care of patients with OSA. *AHI* apnea-hypopnea index, *CPAP* continuous positive airway pressure, *ENT* ear, nose, and throat specialist, *MAD* mandibular advancement device, *OSA* obstructive sleep apnea, *PSG* polysomnography test

A flowchart concerning patient care of patients with OSA is provided in Fig. 1.

In this study, only patients with OSA and AHI > 10 who are dentate were included. Exclusion criteria were AHI < 10, edentulous, (implant or capping) prosthesis, and snoring only.

Out of the group of 629 patients that were referred, a total of 137 patients were considered suitable for this research. In total, 205 patients were discarted because their AHI level was less than ten, 148 patients had no second polysomnography test performed. A total of 139 patients discarded for other reasons (for example: no MAD treatment started or CPAP therapy instead of MAD treatment).

### Mandibular advancement devices

**Fig. 2:**
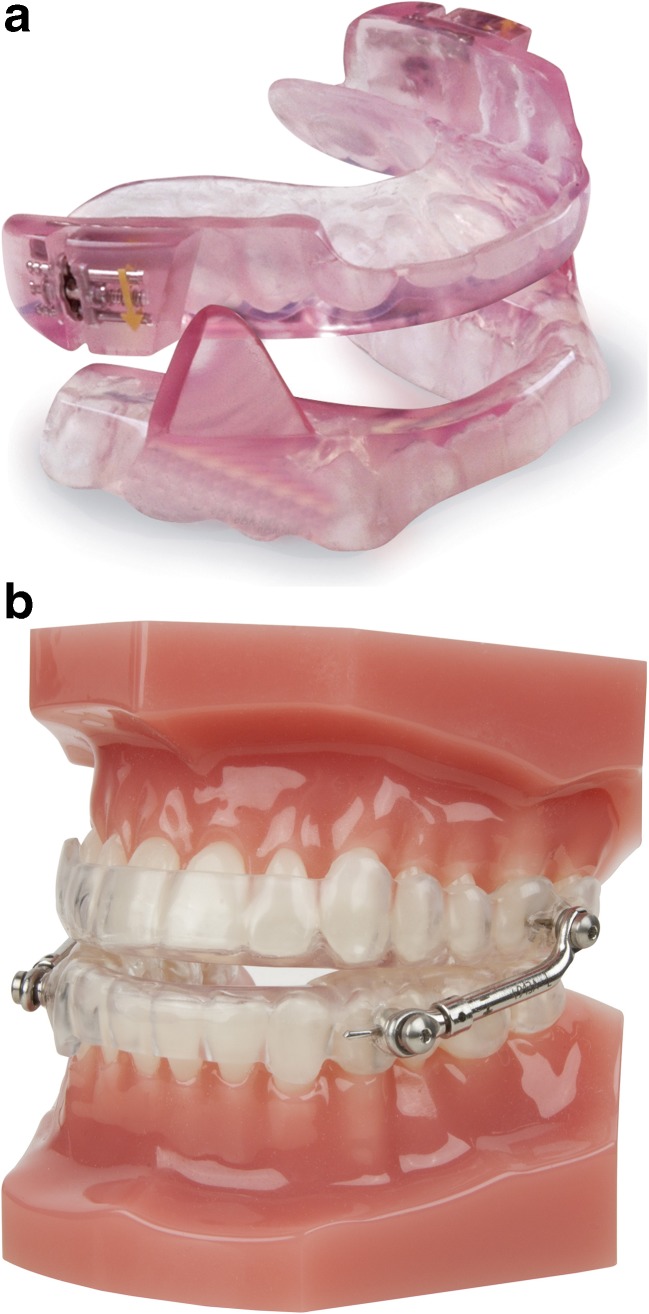
**a** MAD 1 type “Somnodent-Flex,” *MAD* mandibular advancement device. **b** MAD 2 type “Herbst”

Since 2008, two types of MADs are used to treat patients in the MCA. The MADs used are MAD type “Somnodent-Flex,” fabricated by dental laboratory Goedegebuure (MAD 1, Fig. 2a) and MAD type “Herbst,” fabricated by dental laboratory Orthotec (MAD 2, Fig. 2b). Both are acrylic duo bloc titratable oral appliances. MAD type Somnodent-Flex is connected by an adjustable interlocking inclined acrylic buccal extension. MAD type Herbst is connected by Herbst telescoping plunger and tube joints. The alleged difference between MAD 1 and MAD 2 are shown in Table 1.

**Table 1:** Advantages and disadvantages MAD 1 and MAD 2

Type MAD	Advantage	Disadvantage
MAD Somnodent	Less vulnerable	Less freedom of movement (lateral)
	Simple to adjust	Price
	5-year guarantee	
MAD Herbst	Price	More vulnerable
	Less voluminous	No explicit guarantee
	Freedom of movement (lateral)	
	More adjusting possibilities	

*MAD* mandibular advancement device

*MAD* mandibular advancement device

### Variables

During the first 3 months, the MAD can be adjusted for comfort and maximal effectiveness, for each patient individually. The MAD is set at a constant vertical dimension and the patient is instructed how to protrude the MAD. Independent variables: MAD 1 and MAD 2Dependent variable: AHI value.

### Statistical analysis

The data were recorded on a PC using a Microsoft Excel XP database sheet. The statistical analyses are performed using IBM SPSS statistic software for Windows (version 22.0). The data from the excel database sheets were compared by using statistical test techniques.

To compare the means, several techniques, in which independent samples *t* test and paired samples *t* test, were used. The ANCOVA test was used to correct for correlations between means. With the revision of the article, we also performed a sensitivity analysis to determine the minimum inter/intragroup difference/effect size, based on the given sample size, alpha and beta error. Data about the effectivity has been illustrated in scatter plots. Further, we made tables concerning the AHI values on T1 and T2, ∆T1 T2: MAD 1 compared to MAD 2, the correlation ∆T1T2 to T1, and the correlation ∆T2T2 to T1 corrected for T1.

Subgroups and interactions were not present in our study. As it is a retrospective study, there were no missing data. Patients with missing values were excluded. Out of 628 patients, there were 137 patients with all data available selected. There was no loss of follow-up.

## Results

### Patients

Table 2 shows the total of 137 patients that were included in this study. Of the 137 patients, 67 were treated with MAD 1 and 70 patients were treated with MAD 2. Of the 67 patients (MAD 1), 51 were male and 16 female (age 54.51 years, SD ± 10.23 years). Of the 70 patients (MAD 2), 57 were male and 13 female (age 55.59 years, SD ± 9.96 years).

**Table 2:** Overview patient population

	MAD Somnodent	MAD Herbst
Male	51	57
Female	16	13
Total	67	70
Age (years) (SD/range)	54.55 (± 10.24/31–76)	55.5 (± 9.92/31–75)
*p* values	T1 *p* = 0.379 T2 *p* = 0.052	T1 *p* = 0.558 T2 *p* = 0.972

*MAD* mandibular advancement device

For MAD 1, the results of the independent samples test show no significant difference concerning gender, on T1 *t* (65) = 0.887, *p* = 0.379 and on T2 *t* (65) = 1979, *p* = 0.052 (Table 2). For MAD 2, the results also show no significant difference, on T1 *t* (68) = − 0.589, *p* = 0.558 and on T2 *t* (68) = 0.035, *p* = 0.972 (Table 2). Also, no significant differences are found for the mean difference between T1 and T2. Therefore, male and female results are combined.

### Effectiveness MADs: scatter plots

**Fig. 3:**
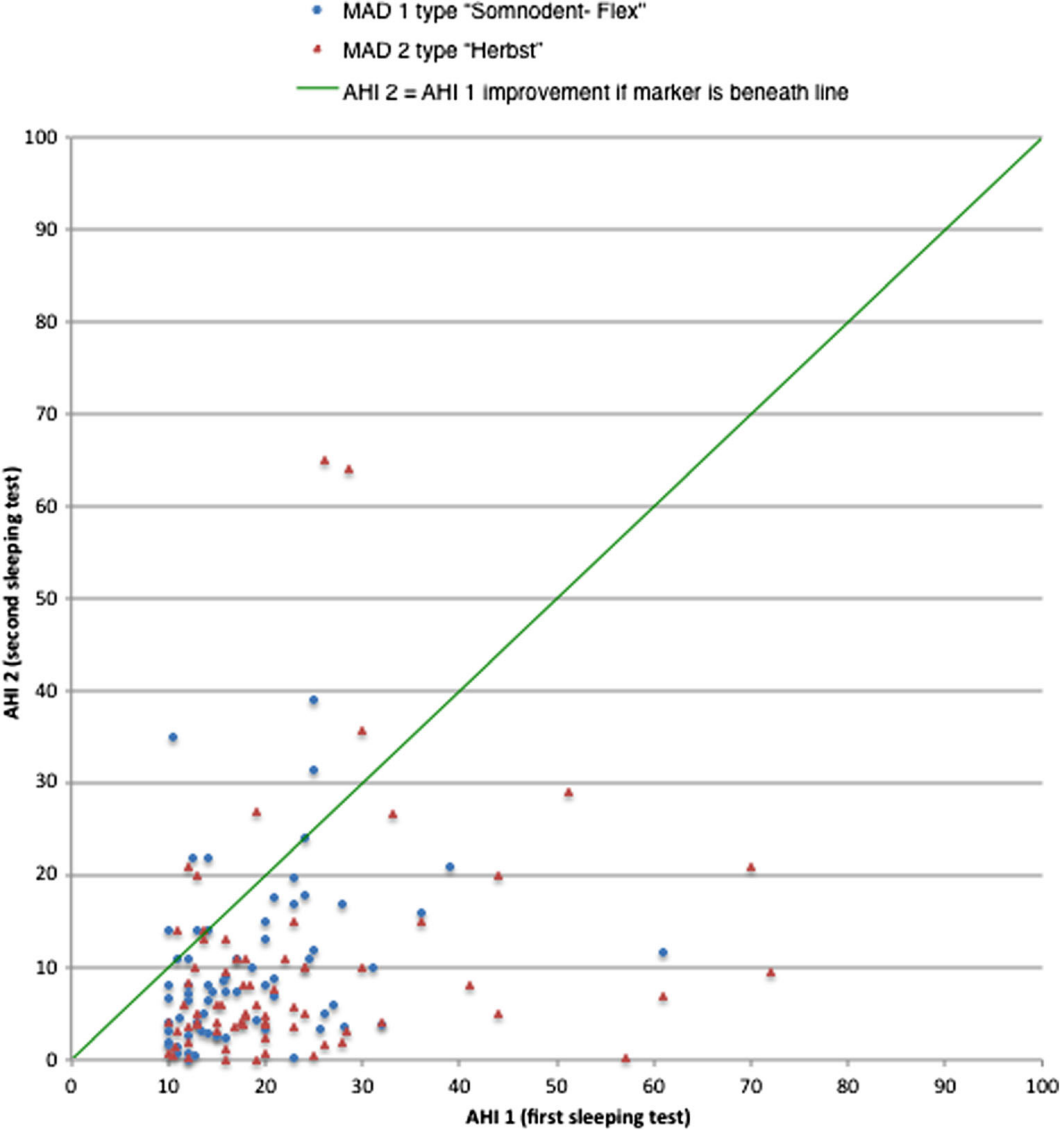
Scatterplot of initial AHI and AHI at the second sleeping test for both types of MAD. *MAD* mandibular advancement device, *AHI* apnea-hypopnea index

In this study, we compare the effectiveness of two types of MAD. First, we test if the two MADs did decrease the AHI. For a global overview, three scatterplots were composed. The scatterplots in Fig. 3 present the AHI values of the both MADs. The *x*-axis presents the AHI of the first sleeping test (AHI 1) and the *y*-axis presents the AHI of the second sleeping test (AHI 2). In the graphs you can see the majority of the dots have a higher AHI 1 value and a lower AHI 2 value, which means for the majority of patients, the AHI decreases during treatment. A few outliers are showing. In these patients the AHI 2 value is higher (in some cases a lot higher) than the AHI 1 value.

### Effectiveness for the different MAD types

The first goal was to determine whether the two MADs decreased the AHI score. A paired *t* test was performed on the two types of MADs to determine if there was a significant difference in their AHI score, on the moment of the first sleeping test (T1) and during the second sleeping test (T2). The results show a decrease in AHI for MAD 1 with a mean AHI of 18.47 on T1 and a mean AHI of 9.61 on T2. For MAD 2, the results also show a decrease in AHI, with a mean AHI of 22.66 on T1 and a mean AHI of 8.80 on T2.

For MAD 1, the paired *t* test shows a significant decrease in AHI occurring from T1 to T2, *t* (66) = 7238, *p* < 0,001. For MAD 2 the paired *t* test also shows a significant decrease in AHI occurring from T1 to T2, *t* (69) = 7736, *p* < 0,001.

### AHI values on T1 and T2

In order to compare the mean AHI’s on T1 to the mean AHI’s on T2 an independent sample *t* test was performed. The results, depicted in Table 3, show a significant difference for MAD 1 compared to MAD 2 on T1 (*p* = 0,035). The mean AHI on T1 for MAD 1 (18.46) is lower compared to the mean AHI on T1 for MAD 2 (22.66).

**Table 3:** Independent samples *t* test for AHI between MAD 1 and MAD 2 on T1 and T2 and for AHI on ∆T1T2

	*N*	T1	T2	∆T1T2
MAD Somnodent	67	18.47 (8. 82)	9.61 (8. 23)	− 8.86 (10.01)
MAD Herbst	70	22.66 (13.66)	8.80 (10.13)	− 13.86 (14.99)
*p* value		*p* = 0.035*	*p* = 0.608	*p* = 0.024*

*p < 0.05

*MAD* mandibular advancement device

The independent samples test shows there is a significant difference in AHI between MAD 1 and MAD 2 on T1, t (135) = − 2125 *p* = 0,035. There is no significant difference in AHI between MAD 1 and MAD 2 on T2, *t* (135) = 0.514 *p* = 0,608 as shown in Table 3.

### ∆T1T2: MAD 1 compared to MAD 2

To determine if the mean difference between T1 and T2 (∆T1 T2) differs similarly for MAD 1 and MAD 2, an independent samples *t* test was performed. The results (Table 3) show the mean difference in AHI for MAD 1 and MAD 2 do differ, *p* = 0.024.

The independent samples *t* test shows a significant difference for ∆T1 T2; *t* (135) = 2288 *p* = 0,024 (Table 3).

### Correlation ∆T1T2 to T1

Because there is a significant difference for both MADs, in ∆T1T2 and in addition the performed independent samples test, (Table 3) showed a ∆T1T2 that relates to T1. Hence, a Pearson correlation test was performed to consider if ∆T1 T2 is correlated to T1. The results show a significant negative correlation for ∆T1 T2; *r* = − 0,726, *p* < 0,001, *N* = 137.

### Correlation ∆T2T2 to T1 corrected for T1

The results show that ∆T1 T2 correlates negatively to T1, which indicates there is a difference on T1 between MAD 1 and MAD 2. To correct for this covariance and the difference on T1 for MAD 1 and MAD 2, an ANCOVA test was performed. With the corrected means an *F* test was performed to see if the variance in AHI for both MADs differs significantly. The results show there is no significant difference between the two groups, MAD 1 and MAD 2, regarding the AHI value after correcting for the covariance; *F* (1, 134) = 140.850, *p* < 0,001; *F* (1, 134) = 1160, *p* = 0.283. The significant difference for ∆T1T2 disappeared through the correction of the means on T1 and T2.

### Power analysis

This study is a retrospective study; therefore, the data were already collected. In this study, an average decrease of 48% in AHI value for MAD 1 and an average decrease of 61% in AHI value for MAD 2 was found. So the difference in effect between the MADs is approximately 13%. A priori, a power analysis, has not been performed.

In this study, an effect size of approximately 13% was found. As a result, a small effect size (*d* = 0.2) is most suitable, compared to the other possible effect sizes, for performing the post hoc power analysis. The post hoc power analysis show that with a small effect size (*d* = 0.2) the power is *p* = 0.2133. With the used sample size in this study we are able to detect effects in 21.33% of the cases. With a medium effect size (*d* = 0.5), the analysis showed a power of *p* = 0.83. If a medium effect size was found, the number of patients in our study would have achieved enough power. However, in this study, we find a small effect size, in this situation the number of patients is too small.

## Discussion

The purpose of this retrospective study was to compare the effectiveness of two types of MADs, MAD “type Somnodent-flex” and MAD “type Herbst” determined by the difference in AHI value (∆T1 T2). ∆T1 T2 turned out to correlate negative to T1. This difference could be caused by a sampling coincidence. Another clarification could be the implementation of the CBO guidelines in 2009. Due to the fact MAD 2 was already in use before the introduction of the guidelines, the patient selection could be altered after the implementation.

With an ANCOVA test, the covariance was corrected. Subsequently, the results show no significant difference between the two groups, MAD 1 and MAD 2, regarding the AHI value. Though, for both MADs, a significant decrease in AHI value was observed. Meaning, both MADs are viable treatment options. The difference in effectiveness of MAD 1 and MAD 2 is approximately 13%. This is a small effect size between MAD 1 and MAD 2.

Regarding the results, we found an optimum therapy response (AHI < 5) in 35.8 and 43.7% of the Somnodent-Flex and Herbst patients, respectively. A good response (AHI < 10) was found in 61.2 and 67.6% of the Somnodent-Flex and Herbst patients, respectively.

As we had a limited sample size, a sensitivity analysis was performed using an independent samples *t* test (on the AHI difference score) with a significance level of 5% and power of 80%, which resulted in an effect size of 0.48. This implies that showing statistical differences (between the devices), smaller than the reported effect size (0.48), is unlikely to occur. However, this effect size of 0.48 is clinically relevant for the patient.

### Results compared with other studies

A recent systematic review and meta-analysis [19] estimated the efficacy of MAD and CPAP. Both appliances were clinically effective in treating OSA. They concluded that MAD treatment is an appropriate option for patients who are intolerant of CPAP and a MAD may be comparable to CPAP in mild apnea.

In the present study, the results showed no significant difference between the two MAD types. For both MADs though, a significant decrease in AHI value was observed. Patients treated with MAD 1 exhibited an average decrease in AHI value of approximately 48% according to baseline, and for MAD 2, the decrease was approximately 61% according to baseline. These results were similar to other studies [13, 17, 18]. In the pilot trial of Aarab et al. (2005), only ten OSA patients participated [13]. They found a short-term effect of approximately 63% decrease in AHI value. Similar effects were found by Dieltjens et al. (2013) who reported an inclusion of 61 OSA patients in his study [17]. They registered a decrease of approximately 62% in AHI value. An average decrease of 57% in AHI value for patients with moderate OSA and an average decrease of 33% in AHI value for patients with severe OSA was found by Vecchierini et al. (2008) [18]. They performed a patient-driven protocol with 40 OSA-diagnosed patients. They registered a decrease of approximately 62% in AHI value.

The interval between the two sleeping tests was at least 3 months, which is a relatively short period. On that account, the results of this study show only a short-term effect. To investigate long-term effects, a longer follow-up period is necessary.

In conclusion of the present study and the studies mentioned, it can be stated that all MAD types give an improvement of the AHI value. The difference between the improvement of the AHI value is small between the types of MAD, especially when the variation is taken into account. It remains questionable whether one or the other type of MAD is superior in effectiveness.

### Reflection on the research method

It can be discussed that the AHI value measured during a sleeping test is only a momentary recording, therefore AHI variability has to be taken into account according to Bittencourt et al. (2001) [20]. Aarab et al. (2009) found similar results reinforcing this finding even further [21]. In this study, a considerable intra-individual variability for four AHI recordings were obtained during a follow-up period of 10 weeks. Although in this study this was not a significant finding, the AHI variability should be taken into account when used to evaluate therapy success.

In this study, we did not take body mass index (BMI) into account. Although several studies have shown that reducing weight could result in a reduction of the severity of OSAS, literature has shown weight loss, or a reduction in BMI, is not a curative treatment method for OSAS but it can support the treatment of OSAS [22].

### Possible clarifications for deviating results

In the scatterplots (Fig. 3), there are a few outliers. These outliers have deviating AHI scores. Sometimes, patients with mainly central apneas or mixed, central, and obstructive apneas, instead of solely obstructive apneas, slip through the selection procedure.

Finally, the impact of the MAD therapy depends on the patient compliance. The results of the second sleeping test can be distorted when the patient compliance, during the first 3 months of optimizing, was poor.

### Quality of life

Unfortunately, due to the nature of the study, only the available data could be taken into account. A standard questionnaire such as the European Social Survey (ESS) has not been used. The ESS collects subjective information about daytime sleepiness and the quality of life of the patients suffering from OSA [6]. By asking questions about personal experiences, such as the ESS, before and during the therapy, it is possible to compare the patient outcomes. At the NWC/MCA, since 2015, a national designed questionnaire, in accordance with the CBO guidelines, is used. The questionnaire is used at the intake and after the second sleeping test.

Based on this study it can be concluded that:

1. MAD treatment decreases the AHI value for patients with an AHI > 10.
2. The type of MAD being type “Somnodent-Flex” or type “Herbst” does not matter.
3. As there is a small effect size, there is need for multicenter prospective clinical trials with a large number of participants.
4. The use of an ESS is advisable in order to elucidate the patients’ subjective opinion on effectiveness.
5. Finally, the diagnosis of OSA is multi-factorial. Therefore, the therapy choice remains very complex and will not always turn out to be the best choice.
